# Clinical Significance of Hepatocyte Growth Factor and Transforming Growth Factor-Beta-1 Levels in Assessing Disease Activity in Inflammatory Bowel Disease

**DOI:** 10.1155/2020/2104314

**Published:** 2020-04-25

**Authors:** Rania Naguib, Wafaa Mohamed El-Shikh

**Affiliations:** ^1^Clinical Science Department, College of Medicine, Princess Nourah Bint Abdulrahman University, Riyadh, Saudi Arabia; ^2^Internal Medicine Department, Endocrinology Unit, Faculty of Medicine, Alexandria University, Alexandria, Egypt

## Abstract

*Background*. Transforming growth factor-beta (TGF-*β*) and hepatocyte growth factor (HGF) are inflammatory cytokines which function as key regulators of immunological homeostasis and inflammatory responses. They have been linked to inflammatory bowel diseases (IBD). In this study, we aim to assess the levels of TGF-*β* and HGF and other inflammatory markers in patients with IBD and correlate them with the disease activity. *Study Design*. A cross-sectional study involving 100 patients with ulcerative colitis (UC) and 100 patients with Crohn's disease (CD) and 50 control subjects. TGF-*β* and HGF levels were measured and correlated with disease activity. *Results and Conclusion*. Serum levels of TGF-*β* and HGF were significantly higher in IBD patients compared with the control group. In the UC group, the levels of HGF and TGF-*β* were significantly higher than in the CD group. Levels of TGF-*β* and HGF correlate with the activity of IBD.

## 1. Introduction

The etiology of inflammatory bowel disease (IBD) is still unknown; however, several mechanisms including inappropriate production of cytokines and dysfunction of the immunological system might play a crucial role. Since the prevalence of IBD is increasing worldwide, it is becoming important to find a noninvasive way to be used as a biochemical marker for early detection of disease exacerbation [1].

In inflammatory bowel disease, the immune response which is responsible for tissue damage is mediated by defects in the counter-regulatory mechanisms which normally suppress the inflammatory response causing repair of mucosal injury. Hepatocyte growth factor (HGF): HGF which is a heterodimer secreted by mesenchymal cells and is found in the lung, liver, intestine, and the central nervous system is one of those mechanisms. It also promotes cell proliferation in vitro. It is also produced by mesenchymal cells during organ injury and stimulates cell division and cell motility. It also promotes normal morphogenic system structure in epithelial cells adjacent to injured areas and helps in the regeneration and repair of damaged tissue. [2]. HGF is known to inhibit inflammatory pathways and has the benefit of reducing the symptoms and the intestinal injury scores of IBD in animal models [3, 4].

Transforming growth factor-*β*1 (TGF-*β*1) is a cytokine that is produced by many cells in the body and targets both immune and nonimmune cells. TGF-*β*1 is produced and secreted from the cells in the lamina propria and the epithelium of the colon. It controls cellular proliferation and takes part in healing and fibrosis [1]. TGF-*β*1acts as a negative regulator of mucosal inflammation, and thus, defective production and/or activity of this biomarker can lead to the development of or exacerbation of colitis [5].

Since the new trends in treating IBD depend mainly on suppression of inflammation, there is a need for better understanding of the potential effects of inflammatory cytokines on the course of the disease. Interaction between genetic and environmental factors leads to immune-pathologic process which promotes chronic inflammation of the gut. This inflammatory process is the major mechanism of Crohn's disease (CD) and ulcerative colitis (UC) which are the major forms of inflammatory bowel disease (IBD) [6]. Recent studies suggest that IBD is caused by an abnormal local immune response to some particles of the bacterial microflora, inappropriately controlled by endogenous counter-regulatory mechanisms [7]. One of these mechanisms involves the production/activity of transforming growth factor-*β* (TGF-*β*).

Many experimental studies have shown that TGF-*β* is considered to be protective to the development of colitis or is associated with the diminished severity of colitis [5] whereas some studies confirm that TGF-*β* is known to play an important role in the pathophysiology of IBD [8]. TGF-*β* promotes the proliferation and differentiation of epithelial cells, and it helps in wound healing and increase in fibrosis during inflammation. It promotes the extracellular matrix production by intestinal cells. Because of the effect on fibrosis, TGF-*β* has also been implicated in stricture formation and muscle hypertrophy which is a common complication in IBD. [9] It has been noted that levels of HGF are increased locally and systemically in case of infection. It was also reported that healing process is accelerated by application of HGF locally to the site of injury in a wound [10]. Growth factors play an important role in the regeneration of injured cells in gastrointestinal organs [2]. HGF is known to decrease IBD manifestations in rat models. It protects the intestinal mucosa by ameliorating the inflammatory response seen so commonly amongst IBD patients [4].

The aim of this study is to assess the plasma levels of HGF and TGF-*β* in patients with IBD to evaluate the level of these markers in both CD and UC patients compared with normal control subjects and to correlate the level of both markers with the disease activity.

## 2. Materials and Methods

A cross-sectional study, purposive sample including 100 patients diagnosed with UC, 100 with CD, and a group of 50 healthy volunteers matched for age and gender were enrolled in this study. Diagnosis of IBD was confirmed by video-colonoscopy and histopathological evaluation of intestinal biopsies. Diagnosis of ulcerative colitis (UC) was based on standard criteria including clinical, radiological, endoscopic, and histological parameters. Inclusion criterion was that not getting treatment for at least 1 month before enrolment. Activity of UC was assessed according to the Mayo scoring system which includes clinical features (stool frequency and rectal bleeding), colonoscopic findings, and physician's global assessment [11]. Endoscopic assessment was performed by colonoscopy using Carbonnel's criteria for moderate and severe colitis [12].

Assessment of disease severity used in the present study included the combined interpretation of patient's general sense of well-being, the performance status, and biological signs of severe disease activity (fever, tachycardia, anemia, accelerated erythrocyte sedimentation rate, and low serum albumin). According to the disease activity index, patients with UC were categorized into severe disease (score ≥9), moderate-severe disease (activity score 6–9 points), or mild disease (activity score 2–5 points) [13].

The activity of Crohn's disease (CD) was measured using Crohn's Disease Activity Index (CDAI), which includes the number of liquid or very soft stools in one week, the sum of seven days abdominal pain ratings, general well-being, symptoms or findings presumed related to Crohn's disease, taking loperamide or opiates for diarrhea, abnormal mass, hematocrit, and weight loss. Patients were classified as having mild disease (activity score 150–220 points), moderate disease (activity score >220–450 points), and severe disease (activity score >450 points).

Patients with inflammatory processes, malignancies, immune disorders, and nutritional abnormalities were excluded. Patients who received immunomodulating drugs at least 6 months prior to the study were excluded. Most IBD (CD and UC) patients were treated with the usual anti-inflammatory drug (5-aminosalicylic acid, 5ASA) at the standard recommended doses. Only minority, with more severe disease course or during the relapse, used immune suppressants (e.g., steroids or azathioprine). The control group included normal blood donors and medical staff of the institution without gastrointestinal complaints or a family history of IBD.

Serum transforming growth factor-*β* levels and hepatocyte growth factor were measured by ELISA using commercial kits (Quantikine TGF-*β*, HGF R&D Systems) following the instructions from the manufacturer.

Statistical analysis: data were analyzed using IBM SPSS statistics 20. Descriptive statistics in terms of means, standard deviations, median, and interquartile ranges were used to describe criteria of the studied sample. Analysis of quantitative data by *t*-test and association of qualitative variables by chi-square test were conducted. Pearson correlation was used to assess the degree of relationship between quantitative variables as appropriate. *p* value less than 0.05 was considered as statistically significant.

Ethical considerations: this study was approved by the local ethics committee for research according to the current institutional guidelines. Informed consent forms of patients and controls were obtained.

## 3. Results

Table 1 shows the demographic characteristics of participants and the degree of disease activity. The level of TFG-*β* was significantly higher in both CD and UC groups compared with the control group (*p*=0.001).

Also, level of HGF in both UC and CD groups was significantly higher than in the control group (*p* < 0.02). The level of TGF-*β* in the UC group was 1423.51 ± 450.5 which was significantly higher than in CD (1016.28 ± 256.07) (*p*=0.001). The level of HGF in the UC group was 1421.30 ± 476.65 which was significantly higher than in CD (1199.21 ± 389.36) (*p*=0.001).

In UC patients, the mean level of TGF-*β* was significantly higher in severe disease compared with moderate disease which was higher than in mild cases. The difference was statistically significant (*p*=0.001).

Also, in CD patients, the mean level of TGF-*β* was significantly higher in severe disease compared with moderate disease which was higher than in mild cases (*p*=0.0011).

In UC patients, HGF was higher in severe degree of disease compared with moderate which was higher than mild severity (*p*=0.0013).

Also, in CD, HGF was higher in severe degree compared with moderate degree which was higher than mild severity (*p*=0.001).

The degree of severity of disease activity in individuals with both Crohn's disease and ulcerative colitis correlated positively with the level of both TGF-*β* and HGF (*p*=0.005), Table 2.

## 4. Discussion

Levels of TGF-*β* and HGF tend to be higher in both CD and UC groups compared with the control group. These results are in concordance with the previous studies [1, 14, 15]. This tendency to the higher TGF-*β*1 and HGF plasma levels in the IBD patients is consistent with the current view that both cytokines exert aggressive effects on pathophysiology events of ulcerative colitis and that TGF-*β* might be the major cytokine during times of active inflammation. Noteworthy, McKaig et al. detected a similar expression of the TGF-*β*1 gene in myofibroblast tissue cultures obtained from inflamed intestinal tissues in both CD and UC patients [16]. Recent studies reported an increased expression of the TGF-*β* gene in intestinal tissue of CD [17] and UC [18] patients compared with controls. On the contrary, Kanazawa et al. studied the expression of TGF-*β* in paraffin-embedded samples from bowel tissue. They examined 11 patients with UC, 11 with CD, and 10 healthy controls. Expression of TGF-*β* in the endothelial cells was not found in either the UC or the CD group [19]. Another study performed on a group of children suffering from chronic inflammatory bowel diseases reported that the TGF-*β* plasma levels showed no significant differences in both CD and UC patients and the control group [20]. Also, there were no statistically significant differences between the TGF-*β* plasma levels in patients with CD and patients with UC in the study done by Kader et al. [21]. An interesting finding was obtained by another study which reported that TGF-*β* was significantly higher than the control in UC patients, while it was lower than the control in CD [22]. These controversary findings might be explained by the altered behavior of cytokines in patients with IBD which might be due to Th1 cell involvement in the immune response in CD patients and Th2 in UC patients [20].

In our study, it was noted increased levels of both HGF and TGF-*β* in UC patients compared with CD patients. These results are similar to results obtained by Wedrychowicz et al. [23] who found increased serum TGF-*β* in UC patients versus the CD group. The difference of levels of TGF-*β* between CD and UC may be attributed to the difference in the degree and extent of the inflammation. Inflammation in CD evolves from superficial into transmural, resulting in deep fissuring ulcers penetrating through the muscle layer, forming fistulas and ulcers, whereas in UC, it is limited to mucosa and submucosa.

In both UC and CD patients, the mean level of both TGF-*β* and HGF was significantly higher in severe disease compared with moderate disease, and it was higher in moderately severe cases compared with mildly severe cases. The degree of severity of both UC and CD correlates positively with the level of both TGF-*β* and HGF. These findings are similar to the results obtained by Ciecko-Michalska et al. [1], while other studies reported no relationship between TGF-*β* levels and the activity UC and CD [19]. In a study done by Kader et al. [21], TGF-*β* was significantly higher in patients with CD in remission than in active disease, while a study done by Kiliç et al. [9] found no relationship between TGF-*β* and disease activity in CD. Another study [20] concluded that the TGF-*β*1 plasma levels showed no correlation with the clinical phase of the disease. These conflicts in findings might be partly explained by rather low severity of the disease at the time of diagnosis in the studied group. It was, therefore, concluded that TGF-*β* and HGF may be key cytokines during periods of active inflammation, modulating epithelial cell restitution, and functional features of cells within the lamina propria.

## 5. Conclusion

The assessment of circulating transforming growth factor-*β* (TGF-*β*) and hepatocyte growth factor (HGF) levels could be a good biologic acute phase response marker of inflammatory bowel disease activity.

## 6. Limitations and Recommendations

In our study, the intestinal tissue levels of TGF-*β*1 and HGF could have been estimated together with the serum level, and the correlation between them could have been studied. Further studies measuring the level of TGF-*β* and HGF in patients with remission and comparing the results with patients with active disease need to be done.

## Figures and Tables

**Table 1:** Age, sex, and degree of disease activity in the CD group and the UC group.

	Groups	
	CD group	UC group	
Age	32.7 ± 11.63	32.68 ± 7.16	*p*=0.322
*Sex*			Total
Female	56 (56.00%)	39 (39.00%)	95 (47.50%)
Male	44 (44.00%)	61 (61.00%)	105 (52.50%)
Total	100 (100.00%)	100 (100.00%)	200 (100.00%)
*Degree of disease activity*			Total
Mild	25 (25.00%)	38 (38.00%)	63 (31.50%)
Moderate	40 (40.00%)	53 (53.00%)	93 (46.50%)
Severe	35 (35.00%)	9 (9.00%)	44 (22.00%)
Total	100 (100.00%)	100 (100.00%)	200 (100.00%)

**Table 2:** Relation between plasma levels of HGF and TGF-*β* and degree of disease activity in the CD group and the UC group.

Degree of disease activity	Mild	Moderate	Severe	*p*
CD group				
HGF range	661.0–999.0	1051.0–1665.0	1890.0–2121.0	
Mean ± S.D.	819.9 ± 87.0	1391.9 ± 161.9	2011.6 ± 91.6	0.001^*∗*^
TGF-*β*				
Range	678.0–1101.0	802.3–1801.0	1101.0–1900.0	
Mean ± S.D.	879.2 ± 115.6	1260.0 ± 231.4	1540.3 ± 364.6	0.001^*∗*^
UC group				
HGF range	703.0–1305.0	1203.0–1611.0	1733.0–2300.0	
Mean ± S.D.	813.2 ± 133.4	1312.8 ± 95.4	1944.6 ± 102.6	0.0013^*∗*^
TGF-*β* range	689.0–1230.0	1226.0–1850.0	1823.0–2398.0	
Mean ± S.D.	797.7 ± 134.8	1618.3 ± 134.9	1998.6 ± 113.7	0.0011^*∗*^

## Data Availability

The data used to support the findings of this study are available from the corresponding author upon request.
